# Drug Hypersensitivity Quality of Life Questionnaire: validation procedures and first results of the Portuguese version

**DOI:** 10.1186/s12955-021-01749-1

**Published:** 2021-05-10

**Authors:** E. Dias de Castro, J. Barbosa, A. M. Mesquita, A. Caires, L. Ribeiro, J. R. Cernadas, I. Baiardini

**Affiliations:** 1 Allergy and Clinical Immunology Department, Centro Hospitalar Universitário de S. João EPE, Porto, Portugal; 2 MedInUP- Center for Drug Discover and Innovative Medicines, Faculty of Medicine, University of Porto, Porto, Portugal; 3 Public Health and Forensic Sciences and Medical Education Department, Faculty of Medicine, University of Porto, Porto, Portugal; 4 UNIC- Cardiovascular Research and Development Unit, University of Porto, Porto, Portugal; 5 Biomedicine Department, Faculty of Medicine, University of Porto, Porto, Portugal; 6 I3S- Instituto de Investigação e Inovação em Saúde, University of Porto, Porto, Portugal; 7 Allergy and Respiratory Diseases Clinic – DIMI, University of Genoa, IRCCS AOU San Martino-IST, Genoa, Italy

## Abstract

**Background:**

Hypersensitivity reactions to drugs are unpredictable and can be very complex and severe, even life threatening. Assess its impact on patient’s health related quality of life (HRQoL) is crucial. The Drug Hypersensitivity Quality of Life Questionnaire (DrHy-Q) is the only validated disease-specific HRQoL questionnaire. We aimed to translate and cross-cultural validate the DrHy-Q to the Portuguese population. It was also our purpose to determine the impact of drug hypersensitivity on patients’ HRQoL.

**Methods:**

The translation and cross-cultural adaptation of the DrHy-Q to Portuguese was performed according to standards. Reliability of the DrHy-Q Portuguese version was assessed in terms of internal consistency and test–retest reliability. Structural validity, divergent validity (with a generic health related QoLQ-PGWBI) and discriminant validity were also evaluated. Forty patients accepted to participate in the validation phase. The Portuguese version of the DrHy-Q was applied to 260 consecutively adult patients, studied in our Department for suspected drug hypersensitivity.

**Results:**

The Portuguese DrHy-Q showed adequate internal consistency (Cronbach’s ɑ = 0.938), good test–retest reliability [ICC = 0.713 (95% CI 0.488–0.850] and one-dimensional structure. No significant correlation was found between the DrHy-Q and the PGWBI total scores (r = − 0.010, *p* = 0.957). Two hundred of patients completed the study: 78.5% female; mean age = 44 ± 15 years. Mean DrHy-Q score was 36.8 ± 12.6. Two clinical factors significantly predict DrHy-Q total score: clinical manifestations and number of suspected drugs. Patients with anaphylaxis (β = 11.005; 95% CI 5.523; 16.487), urticaria/angioedema (β = 7.770; 95% CI 2.600; 12.940) and other manifestations (β = 7.948; 95% CI 1.933; 13.962) are more likely to have higher DrHy-Q total score than patients with maculopapular exanthema. Patients with ≥ 2 suspected drugs are also more likely to have worse QoL (β = 7.927; 95% CI 3.687; 12.166).

**Conclusion:**

The Portuguese version of DrHy-Q revealed adequate validity and reliability, indicating that it is appropriate to assess the impact of drug hypersensitivity on patients’ HRQoL, providing data for a better comprehension and management of our patients. Moreover, our results highlight that the severity of the drug hypersensitivity reaction and the number of suspected drugs have impact on patient’s DrHy-QoL.

**Supplementary Information:**

The online version contains supplementary material available at 10.1186/s12955-021-01749-1.

## Background

Drug allergic reactions are adverse drug reactions mediated by immunological mechanisms. They are unpredictable and potentially severe, even life-threatening. In practice, when an immunological mechanism cannot be demonstrated, the term drug hypersensitivity is applied to adverse drug reactions that clinically resemble allergy. Drug hypersensitivity reactions (DHR) comprise 15% of all adverse drug reactions [1–3] and are responsible for significant morbidity and socio-economical costs [4]. Due to their unpredictable nature and potential severity, drug hypersensitivity may have an important impact on patients’ health-related quality of life (HRQoL). HRQoL is a central patient reported outcome defined as those aspects of self-perceived well-being that are related to or affected by the presence of disease or treatment [5].

The assessment of HRQoL becomes critical in routine clinical practice, research and regulatory processes, namely in allergic and other chronic diseases. It allows a broader understanding of the impact on patients of their diseases and therapies [6, 7], regardless any interpretation by physician or others [8]. However, HRQoL is rarely assessed in patients with DHR probably due to the lack of specific tools for this purpose [4, 9]. The only specific tool measuring HRQoL in this field is the Drug Hypersensitivity Quality of Life Questionnaire (DrHy-Q) developed and validated by an Italian group [6]. The DrHy-Q was derived from a 34 generation-phase items to a 15-items questionnaire. The purpose was to assess the specific burden of drug hypersensitivity from the patient’s perspective, specifically the impact of the drug hypersensitivity in the patient’s life from emotional, physical and social point of view. This questionnaire was shown to be suitable for assessing quality of life in patients that experienced a DHR and can be used alone or in combination with other patient-reported outcome questionnaires. Subsequently, the DrHy-Q was translated and validated in some cultural settings such as Spain [9, 10], Turkey [8], Netherlands [4] and Thailand [11], but not in Portugal or in the Portuguese language.

The main aim of this study was to translate and cross-cultural validate the original Italian version of the DrHy-Q to the Portuguese population. The secondary aims were to determine the impact of drug hypersensitivity on patients’ HRQoL and to compare the clinical features between patients with confirmed versus excluded drug hypersensitivity.

## Methods

We used the only validated health related quality of life questionnaire in the field of drug allergy, the Drug Hypersensitivity Quality of Life Questionnaire (DrHy-Q). It is a 15-item tool, self-completed and evaluated on a five-point Likert scale [from 1 (not at all) to 5 (very much)]. Higher scores signify worse HRQoL [6].

### Translation and cross-cultural adaptation of DrHy-Q

The translation and cross-cultural adaptation of this HRQoL to the Portuguese language was performed according to the standards for translation and cultural adaptation for Patient-Reported Outcomes (PROs) tools [7, 12, 13].

#### Forward translation

After obtaining authorization from the authors of the original instrument, the DrHy-Q [6] was translated to Portuguese language. This phase involved two local translators (Portuguese native speakers, familiar with the specific terminology and knowledgeable of the italian-speaking culture), a Portuguese HRQoL expert and a Portuguese Allergy specialist and included the following steps:

- Independently production of 2 forward versions of the questionnaire (original items, instructions and response choices), by each translator (Version 0a and Version 0b);
- Production of a single reconciled version by both translators and researchers, after discussion of the 2 forward versions (Version 1).

#### Backward translation

In this phase, the first reconciled forward version of the questionnaire (Version 1) was translated back into the source language by a local translator (native speaker of the target language and bilingual in source language) with no access to the original source version of the questionnaire. This backward translation, was sent to the authors in order to be compared with the original source version and detect any misunderstandings, mistranslations or inaccuracies. After the authors review and as result of this phase, a second version of the questionnaire was obtained (Version 2). Only 1 minor change was made from the first to the second version.

#### Pilot testing

In order to determine if the translated questionnaire is understood and the language simple and appropriate, the second version of the questionnaire was tested on a panel of 30 adult patients, through face to face interviews conducted by the same interviewer (Allergy specialist). Patients were inquired regarding any difficulty in understanding the questionnaire and their interpretation of all items was checked. This pretest phase showed that the questions were easily understandable and do not require explanation. Patients suggested only a minor change to item 2, in order to optimize the understanding. As a result of this phase, a third and final version of the translated questionnaire was produced (“Appendix”).

### Validation procedures

The psychometric properties of the Portuguese version of the DrHy-Q were evaluated in accordance with current guidelines [14, 15]. Data was collected using a convenience sampling method [16]. A randomized group of 40 patients accepted to participate in this validation phase.

#### Reliability

Reliability refers to the consistency of the measure. Internal consistency and test–retest reliability were assessed.

The test–retest reliability of the DrHy-Q was evaluated by applying the questionnaire to the randomized group of 40 patients on two different occasions, one-week apart, without any intervention, personal or clinical significant changes. Questionnaires were sent by e-mail.

#### Validity

Validity is the degree to which an instrument measure what is intended to measure. The validity of the DrHy-Q was assessed with 3 different analyses: structural validity, divergent validity and discriminant validity.

Structural validity was assessed with exploratory factor analysis (EFA) [17]. The PGWBI instrument was chosen for the purpose of ascertaining divergent validity. It is expected a lack of correlations between PGWBI and HRQoL since theoretically they measure different constructs. The 40 randomized patients involved in the validation phase were also asked to complete the Portuguese validated version of the Psychological General Well-being Index (PGWBI) [18]. The PGWBI is a brief self-administered questionnaire with 22 items on a six-point Likert scale. Each item is scored from 0 to 5 points, leading to a total score between 0 and 110 points, with higher values indicating better well-being [19]. The PGWBI evaluates six mood states (anxiety, depressed mood, positive well-being, self-control, general health, and vitality) and has been extensively used as an indicator of HRQoL in patients with chronic conditions [6–8]. Discriminant validity, the instrument’s ability to differentiate between populations that are known or expected to differ [6], was evaluated by comparing DrHy-Q scores from patient groups with different clinical characteristics.

### Drug diagnostic work-up

The drug hypersensitivity diagnostic work-up was performed according to international guidelines [1], in order to confirm or exclude the suspected drug hypersensitivity. The type and severity of reaction, the suspected drug and the availability of validated skin tests and *in-vitro tests* were considered. It included a validated questionnaire (the drug hypersensitivity questionnaire developed by European Network of Drug Allergy- ENDA) [20] and, depending on the previous referred factors, skin tests (prick, intradermal and/or patch) [21, 22], specific IgE and drug provocation test (DPT) [23].

The diagnosis of drug hypersensitivity was confirmed when skin tests were positive for validated concentrations or when specific provocation test with the suspected drug was positive. On the other hand, drug hypersensitivity diagnosis was excluded if all diagnostic procedures, including DPT with the suspected drug, were negative. Patients that did not complete the diagnostic work-up or that did not reach a conclusive diagnosis were excluded.

### Study population

The final version of the translated questionnaire was applied, from July 2017 to December 2018, to 260 consecutively patients ≥ 18 years-old, studied in our Allergy Department for suspected drug hypersensitivity. The patients filled the questionnaire in their first visit to the day hospital.

Individuals that do not completed the DrHy-Q were excluded.

Social-demographic data were also collected.

Written informed consent was obtained by all individuals included in the study and the protocol was approved by the Hospital Ethics Committee (CE-66-2016).

### Statistical analysis

Data were entered and analyzed using the Statistical Package for Social Sciences version 25 and statistical significance was defined as *p* < 0.05.

Variables were expressed as mean ± standard-deviation or relative and absolute frequencies. Categories with few cases were combined.

Internal consistency of the DrHy-Q was tested by Cronbach’s alpha coefficient. A value of ≥ 0.70 is considered adequate for group comparison, but a value of 0.90–0.95 is needed for clinical application [24–26].

The test–retest reliability of the DrHy-Q was evaluated by applying the questionnaire to the randomized group of 40 patients on two different occasions, as described above. The 2 score sets were analyzed by the *t* test for paired samples [26]. The intra-class correlation coefficient (ICC); a two-way mixed model, was also used to assess test–retest reliability [27]. This model indicates the proportion of variance that is due to between-subject variability relative to the sum of between-subject variability and measurement error. Criteria for ICC values were < 0.4 as poor, 0.4–0.59 as fair, 0.6–0.74 as good and > 0.75 as excellent reliability.

The Bland–Altman plot was also created to visualize the agreement between the questionnaire responses at the two time points (baseline and retest) [28].

The validity of the structure was tested using the EFA [17]. In order to evaluate the number of dimensions that emerged from the data EFA with maximum likelihood was performed. To determine the number of factors to be included eigenvalues > 1 and the scree plot were examined. Items with factor loadings below 0.40 were planned to be eliminated.

To assess divergent validity, the Pearson correlation was used to analyze the correlation between the DHRQoL and the PGWBI total and the 6 domain scores. Values of − 0.25 to 0 mean poor or no correlation; − 0.50 to − 0.25 = fair; − 0.75 to − 0.50 = moderate-to-good; − 1.0 to − 0.75 = good-to-excellent correlation.

Independent t-test or ANOVA were used to analyze differences between groups on DrHy-Q total score.

Additionally, a multiple linear regression model was designed using automatic linear modeling (ALM) [29] to evaluate which factors are associated with DrHy-Q total score. Automatic Linear Modeling is a modification and improvement of the traditional linear regression procedure, particularly in automatic variable selection and automatic data preparation [29] Multiple combinations and weighted importance are tested to determine the optimum and best-fit model possible [30]. This method also ranks predictors according to their degree of dependence from the less important to the most important.

Significant variables in the bivariate analysis were included in the model. The forward stepwise approach was used and Akaike Information Criterion Corrected (AICC) was selected as the variable entry/removal criterion. These analyses assess the discriminant validity of the DrHy-Q.

## Results

### Study population and Drug allergy work-up

Two hundred of patients completed the diagnostic work-up and fully filled the DrHy-Q, during the study period: 78.5% were female with a mean age of 44 ± 15 years, range [18–77]. All data in the Additional file 1.

There were more 60 patients studied during this period that were excluded: 6 patients did not fully complete the DrHy-Q; 29 did not complete the drug allergy diagnostic work-up or did not have a conclusive result and 25 patients missed the identification number in the DrHy-Q and could not be linked to the drug diagnostic work-up. These patients were not included in any analysis.

Antibiotics were the most commonly implicated drugs (168/220 patients, 84%), followed by Nonsteroidal Anti-inflammatory drugs-NSAID (44/200 patients; 22%). The main clinical presentations were maculopapular exanthema-MPE (39.5%), urticaria/angioedema (22.5%) and anaphylaxis (20%). Fifty-one percent of the reactions were immediate and 39.5% non-immediate.

After completing the drug allergy work-up 93 patients (46.5%) had the final diagnosis of confirmed drug allergy/hypersensitivity.

The likelihood of confirmed drug hypersensitivity versus a negative diagnostic work-up was significantly higher in the cases involving immediate reactions (66.3% vs 39%, *p* < 0.001), severe reactions (56.5% vs 1.5%, *p* < 0.001), anaphylaxis (40.0% vs 4%, *p* < 0.001), higher number of reactions (1.7% vs 1.3% *p* < 0.001), higher number of suspected drugs (1.5% vs 1.3% *p* = 0.006), NSAID (37.6% vs 8.4%, *p* < 0.001), general anesthetics (12.9 vs 2.8, *p* = 0.008) and an interval of time of ≤ 2 years between reaction and study (44.0% vs 25.0% *p* = 0.027).

Table 1 summarizes the socio-demographic and clinical characteristics of the patients and differences between patients with confirmed drug hypersensitivity and those with excluded drug hypersensitivity. There are some missing data mainly concerned to the socio-demographic characteristics.

**Table 1:** Socio-demographic and clinical characteristics of patients

	Confirmed drug hypersensitivity (N = 93)	Excluded drug hypersensitivity (N = 107)	*p* value
Gender, n (%)			
Female	74 (79.6)	83 (77.6)	0.863
Male	19 (20.4)	24 (22.4)	
Age in years, Mean (SD)	46.0 (15.0)	43.0 (15.0)	0.195
Academics studies, n (%)			
≤ 6 years	4 (11.4)	10 (24.4)	0.325
7–12	16 (45.7)	18 (43.9)	
> 12	15 (42.9)	13 (31.7)	
Atopy, n (%)			
Yes	30 (33.3)	28 (26.7)	0.347
No	60 (66.7)	77 (73.3)	
Co-morbidities, n (%)			
Yes	59 (63.4)	58 (54.7)	0.249
No	34 (36.6)	48 (45.3)	
Daily medication, n (%)			
Yes	58 (62.4)	55 (51.9)	0.153
No	35 (37.6)	51 (48.1)	
Family history of drug allergy, n (%)			
Yes	5 (5.5)	7 (6.6)	0.776
No	86 (94.5)	99 (93.4)	
Δ reaction- study, n (%)			
≤ 2 years	33 (44.0)	23 (25.0)	**0.027**
> 2 and ≤ 5 years	21 (28.0)	29 (31.5)	
> 5 years	21 (28.0)	40 (43.5)	
Type of reaction, n (%)			
Immediate	61 (66.3)	41 (39.0)	**< 0.001**
Non-Immediate	24 (26.1)	55 (52.4)	
Unknown	7 (7.6)	9 (8.6)	
Clinical manifestations, n (%)			
Anaphylaxis	36 (40.0)	4 (3.9)	**< 0.001**
Urticaria/Angioedema	24 (26.7)	21 (20.6)	
Maculopapular exanthema	25 (27.8)	54 (52.9)	
Cardiovascular symptoms	0 (0.0)	8 (7.8)	
Bronchospasm	2 (2.2)	2 (2.0)	
Gastrointestinal symptoms	0 (0.0)	3 (2.9)	
Fixed drug eruption	1 (1.1)	2 (2.0)	
Unspecified general symptoms	2 (2.2)	8 (7.8)	
Severity of reaction n (%)			
Mild	5 (21.7)	142 (72.1)	**< 0.001**
Moderate	5 (21.7)	52 (26.4)	
Severe	13 (56.5)	3 (1.5)	
Number of reactions, Mean (SD)	1.7 (1.0)	1.3 (0.5)	**< 0.001**
Number of suspected drugs, Mean (SD)	1.5 (0.8)	1.3 (0.5)	**0.006**
Suspected drug, n (%)			
ßLantibiotics	55 (59.1)	78 (72.9)	0.051
Non-ßLantibiotics	14 (15.1)	21 (19.6)	0.458
NSAID	35 (37.6)	9 (8.4)	**< 0.001**
General anesthetics	12 (12.9)	3 (2.8)	**0.008**
RCM	9 (9.7)	6 (5.6)	0.296
Corticosteroids	7 (7.5)	3 (2.8)	0.193
Local anesthetics	1 (1.1)	7 (6.5)	0.07
PPI	5 (5.4)	2 (1.9)	0.254
Other	9 (9.8)	3 (2.8)	0.069

Bold values indicate statistical significance (*p* < 0.05)

SD, standard deviation; Δ reaction- study, interval of time between reaction and study; ßL, beta-lactam; NSAID, non- steroidal anti-inflammatory drugs; RCM, radiocontrast media; PPI, proton pomp inhibitors

### Validation procedures

Thirty-one patients fully completed the questionnaires applied at the validation (83% were female with a mean age of 39 ± 12 years) (Additional file 2).

#### Reliability

The questionnaire’s global internal consistency was 0.938, adequate not only for group comparison but also for clinical application.

**Fig. 1:**
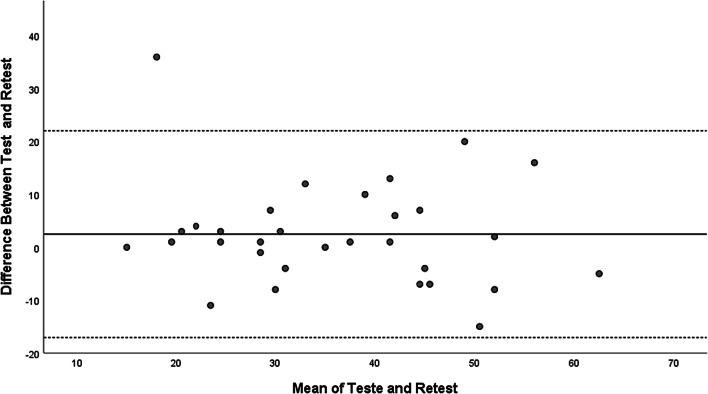
The Bland–Altman plot: visualization of the questionnaire responses at the two time points (baseline and retest)

No significant differences were found between test and retest (*p* = 0.176) with a mean difference of 2.5 (9.9) and a correlation of 0.722 (*p* < 0.001). The single measure of ICC was 0.713 (95% CI 0.488–0.850) indicating good test–retest reliability. The Bland–Altman plot indicate no systematic bias (Fig. 1). Only one participant fell outside the expected limits of ± 2SD (− 17.1 to 22.0).

#### Validity

The DrHy-Q Portuguese revealed a one-dimensional structure that explained up to 54.5% of the total variance. Table 2 provides items’ factor loadings. All items had a factor loading above 0.40, therefore, none was excluded.

**Table 2:** Factor loading of each item in Portuguese version of DrHy-Q

Item	Factor loading
Since I am unable to take drugs every illness limits me more than other	0.742
I am afraid of being administered a drug during an emergency to which I am allergic	0.673
I feel frightened due to my problem of allergy reaction	0.807
The problem of adverse reaction to drugs affects my life	0.826
I would like the allergist’s opinion before taking drugs prescribed by other specialists	0.666
Even a little discomfort for me is a problem	0.720
The fact that I cannot use medication safely made me feel different from others	0.735
I feel anxious due to my problem of allergy reaction	0.864
For each disease I would be confident that there is a drug that I can safely take	0.684
I am afraid I could not deal with the pain	0.558
I feel anguished due to my problem of allergy reaction	0.838
I worry every time I take a drug different from ones that cause my allergic reactions	0.812
I give up leisure activities (sport, vacations, trips) because of my problem	0.551
I’m in a bad mood due to my problem of allergy reaction	0.714
The idea of taking a medicine makes me feel anxious	0.791

Concerning divergent validity, no significant correlation was found between the DrHy-Q and the PGWBI total scores (r = − 0.010 *p* = 0.957). Only a fair and negative correlation was observed between DrHy-Q and the dimension general health of the PGWBI (r = − 0.490; *p* = 0.006), but no correlation with the other 5 dimensions (Table 3).

**Table 3:** Pearson’s correlation between the DrHy-Q and the PGWBI domains

PGWBI domain	r	*p*
Well being	− 0.059	0.757
Anxiety	− 0.300	0.107
Depression	− 0.178	0.346
Self-control	− 0.131	0.491
General health	− 0.490	**0.006**
Vitality	− 0.088	0.644
PGWBI total	− 0.010	0.957

Bold value indicates statistical significance (*p* < 0.05)

DrHy-Q, Drug Hypersensitivity Quality of Life Questionnaire; PGWBI, Psychological General Well-Being Index

### DrHy-Q results

In the Table 4 are represented the results of the DrHy-Q, namely the total score according to social-demographic and clinical characteristics of patients. Mean DrHy-Q score was 36.8 ± 12.6 (with median 37.0; minimum: 14.0; maximum 68.0). The following factors were found to be significantly associated with higher DrHy-Q total score or impaired quality of life: older age; presence of co-morbidities; daily medication; absence of family history of drug allergy; clinical manifestations such as anaphylaxis, urticaria/angioedema or other manifestations; severe reactions; ≥ 2 drug reactions; ≥ 2 suspected drugs and allergy to ßL-antibiotics.

**Table 4:** DrHy-Q total scores by socio-demographic and clinical characteristics of patients

	N	(%)	Mean	SD	*p* value
Gender					
Female	157	(78.1)	36.8	12.3	0.95
Male	44	(21.9)	36.9	12.7	
Age					
≤ 38 years	69	(34.3)	32.4	10.5	**0.001**
39–51	68	(31.8)	38.1	13.1	
> 51	64	(31.8)	40.2	13.0	
Academics studies					
≤ 6 years	14	(18.4)	41.9	11.5	0.058
7–12	34	(44.7)	33.2	11.7	
> 12	28	(36.8)	34.2	11.3	
Atopy					
Yes	58	(29.6)	38.9	13.2	0.120
No	138	(70.4)	35.9	12.1	
Co-morbidities					
Yes	118	(59.0)	38.5	13.3	**0.025**
No	82	(41.0)	34.4	11.3	
Daily medication					
Yes	114	(57.0)	39.1	13.0	**0.002**
No	86	(43.0)	33.7	11.4	
Family history of drug allergy					
Yes	12	(6.1)	29.8	11.1	**0.047**
No	186	(93.9)	37.2	12.6	
Δ reaction- study					
≤ 2 years	56	(33.3)	37.2	13.3	0.216
2 e 5	50	(29.8)	33.4	7.9	
> 5	62	(36.9)	36.8	14.2	
Type of reaction					
Immediate	102	(51.5)	38.3	13.5	0.132
Non-Immediate	80	(40.4)	34.6	10.9	
Unknown	16	(8.1)	38.3	14.5	
Clinical manifestations					
Anaphylaxis	79	(40.9)	41.0	13.4	**0.002**
Urticaria/Angioedema	40	(20.7)	39.2	12.5	
MPE	46	(23.8)	32.7	10.2	
Other	28	(14.5)	36.9	13.6	
Severity of reaction					
Severe	45	(23.3)	41.1	13.8	**0.008**
Moderate	58	(30.1)	36.9	12.1	
Mild	90	(46.6)	34.1	11.2	
Number of reactions					
1	127	(65.1)	34.4	12.8	**0.002**
≥ 2	68	(34.9)	40.3	11.1	
Number of suspected drugs					
1	140	(70.4)	35.2	13.0	**0.014**
≥ 2	59	(29.6)	39.9	10.4	
Class of drug					
ßL antibiotics					**0.009**
Confirmed	46	(34.6)	39.3	12.7	
Excluded	87	(65.4)	33.5	11.8	
Non-ßL antibiotics					0.067
Confirmed	10	(28.6)	41.0	11.6	
Excluded	25	(71.4)	33.8	9.4	
NSAID					0.820
Confirmed	26	(65.0)	42.9	13.1	
Excluded	14	(35.0)	41.9	13.1	
RCM					0.324
Confirmed	7	(46.7)	33.9	12.0	
Excluded	8	(53.3)	40.4	12.5	

Bold values indicate statistical significance (*p* < 0.05)

SD, standard deviation; MPE, Maculopapular exanthema; DH, Drug hypersensitivity

Comparing DrHy-Q scores of patients with confirmed drug allergy versus excluded drug allergy we observed significant higher values in the former group (40.5 ± 12.9 vs 33.4 ± 11.2, *p* < 0.001).

Significant variables in the bivariate analysis were included in the multiple linear regression model. Two clinical factors were identified to significantly predict DrHy-Q total score: clinical manifestations and number of suspected drugs.

Patients with anaphylaxis (β = 11.005; 95% CI 5.523; 16.487), urticaria/angioedema (β = 7.770; 95% CI 2.600; 12.940) and other manifestations (β = 7.948; 95% CI 1.933; 13.962) are more likely to have higher DrHy-Q total score than patients with MPE. Patients with two or more suspected drugs are more likely to have higher DrHy-Q total score (β = 7.927; 95% CI 3.687; 12.166), as shown in Table 5. These results support the discriminant validity of the DrHy-Q.

**Table 5:** Predictive factors of DrHy-Q total score

Variable	Β	*p* value	CI 95%	Importance^a^
Clinical Manifestations				0.612
Anaphylaxis	11.005	< 0.001	5.523; 16.487	
Urticaria/angioedema	7.770	0.004	2.600; 12.940	
Other manifestations	7.948	0.010	1.933; 13.962	
MPE	Ref			
Number of drugs				0.388
≥ 2	7.927	< 0.001	3.687; 12.166	
1	Ref			

MPE, maculopapular exanthema

^a^Measure of the relative importance of each predictor in estimating the model

## Discussion

This study showed that the Portuguese version of DrHy-Q is a reliable and valid instrument for assessing HRQoL in patients with drug hypersensitivity.

The statistical analyses confirmed that Portuguese version of the DrHy-Q met the standards for adequate internal consistency with a Cronbach’s alpha of 0.938 (Italian version 0.928) [6] along with a good test–retest reliability.

Factor analysis revealed a one-dimensional structure in line with the original Italian questionnaire^5^ and other translated versions [4, 8, 11].

As expected, a negative but no significant correlation was found between the DrHy-Q and the PGWBI total scores r = − 0.010 *p* = 0.957, since the DrHy-Q is a disease-specific questionnaire (higher scores indicating worse HRQoL) and the PGWBI is a generic quality-of-life questionnaire (higher scores indicating better well-being). Only a fair and negative correlation was observed between DrHy-Q and the dimension general health of the PGWBI (r = − 0.490; *p* = 0.006), but no statistically significant correlation with the other 5 dimensions. These results suggest that the Portuguese DrHy-Q not only measures specific aspects of HRQoL in patients with drug hypersensitivity not assessed with generic tools, but also partially captures general aspects of HRQoL. So, these results, with slight differences with the other studies [4, 6, 8, 10, 11], corroborate an adequate divergent validity. The Italian study [6] reported negative weak correlations and other studies negative fair correlations [4, 8, 11]. A negative significant correlation for the depressed mood dimension of the PGWBI questionnaire and the Spanish DrHy-Q (r = − 0.531; *p* = 0.016) was reported, but not with other dimensions [10].

The Portuguese DrHy-Q was able to discriminate patients according to some clinical aspects. Patients with anaphylaxis, urticaria/angioedema and other manifestations are more likely to have higher DrHy-Q total score than patients with MPE. Patients with two or more suspected drugs are also more likely to have higher DrHy-Q total score. These results are in accordance with other studies: Italian DrHy-Q also found worst HRQoL (higher scores) in patients with anaphylaxis than in patients with other milder reactions [6, 31] and the Dutch DrHy-Q was able to distinguish between patients with one and patients with more than one implicated drug [4]. The Turkish DrHy-Q could discriminate patients with ≥ 2 drug hypersensitivity reactions and patients with only one reaction, and also patients with drug-induced respiratory reactions and those without respiratory reactions [8]. The Thai DrHy-Q was able to distinguish patients with or without life-threatening severe adverse cutaneous reactions (SACRs), patients with reaction to either NSAID or ßL antibiotics, or both, and patients with one or multiple implicated drug classes [11]. Our study also showed an impaired HRQoL in patients with ≥ 2 drug hypersensitivity reactions and patients with allergy to ßL antibiotics, but this variable lost significance in multivariate model. As only 4 patients referred isolated respiratory symptoms they were included in the group of other manifestations. Patients with SCARs were not included since the severity of the reaction contraindicate the usual “in-vivo” allergy diagnostic procedures.

One of the strengths of the current study was the assessment of the potential role of socio-demographic and clinical data in the overall results DrHy-Q. We could observed how some clinical aspects of the DHR could influence the DrHy-Q scores.

Another interesting aspect of this study was the correlation between DrHy-Q scores and the final result of diagnostic work-up. Comparing DrHy-Q scores of patients with confirmed drug hypersensitivity versus excluded drug hypersensitivity we observed significant higher values in the former group. We must point out that the participants filled the questionnaire before any intervention, so they did not know any result of diagnostic work-up. These results were probably explained by the fact that some clinical variables associated with a higher likelihood of confirmed drug hypersensitivity were also associated with higher DrHy-Q scores.

The present study only included patients studied in the day hospital for suspected drug hypersensitivity and this is one limitation, as left out patients with severe adverse cutaneous reactions (SCARs), for instances. However, these are very rare drug hypersensitivity clinical presentations, particularly in our population.

Additionally, the majority of the patients included in this study had antibiotic and/or NSAID hypersensitivity, reflecting the global prevalence of the class of drugs involved in drug hypersensitivity, but limiting the possibility of assessing the impact of hypersensitivity to other class of drugs in patients’ HRQoL.

Finally, our study design did not allow us to explore the sensitivity of DrHy-Q to changes after interventions (e.g. causal diagnosis or desensitization). In future, we intend to assess the responsiveness of the DrHy-Q to interventions trough a larger and multicenter study.

## Conclusions

The Portuguese version of DrHy-Q revealed adequate validity and reliability, indicating that it is appropriate to assess the impact of drug hypersensitivity on patients’ HRQoL, providing data for a better comprehension and management of our patients.

Moreover, our results highlight that the severity of the drug hypersensitivity reaction and the number of suspected drugs have impact on patient’s DrHy-QoL.

### Supplementary Information

**Additional file 1:** Patient database: results of the drug hypersensitivity study and DrHy-Q.

**Additional file 2:** Database on the validation psychometric properties of the DrHy-Q.

## Data Availability

Provided as a supplement to manuscript.

## Appendix: Questionário

**Table:** As reações alérgicas a medicamentos podem influenciar o bem-estar físico e psíquico da pessoa. Solicitamos que indique as dificuldades que tem devido a este problema

		Nenhumas	Poucas	Bastantes	Muitas	Muitíssimas
1	Como não posso tomar qualquer medicamento, qualquer doença me limita mais do que às outras pessoas	1	2	3	4	5
2	Tenho receio de que, durante uma situação urgente, me administrem um medicamento ao qual sou alérgico	1	2	3	4	5
3	Sinto-me assustado, devido ao meu problema de alergia a medicamentos	1	2	3	4	5
4	O problema de alergia a medicamentos afeta a minha vida	1	2	3	4	5
5	Gostaria de ter a opinião de um Imunoalergologista, antes de tomar um medicamento prescrito por outros especialistas	1	2	3	4	5
6	Mesmo um pequeno desconforto é um problema para mim	1	2	3	4	5
7	O fato de não poder tomar tranquilamente qualquer medicamento faz-me sentir diferente dos outros	1	2	3	4	5
8	Sinto-me ansioso, devido ao meu problema de alergia a medicamentos	1	2	3	4	5
9	Gostaria de ter a certeza de que, para cada doença, há um medicamento seguro para mim	1	2	3	4	5
10	Tenho receio de não conseguir lidar com a dor	1	2	3	4	5
11	Sinto-me angustiado, devido ao meu problema de alergia a medicamentos	1	2	3	4	5
12	Fico preocupado sempre que tomo um medicamento mesmo que seja diferente daqueles que me causaram reação alérgica	1	2	3	4	5
13	Desisti de atividades de lazer (desporto, férias, viagens) devido ao meu problema	1	2	3	4	5
14	Sinto-me com a moral em baixo, devido ao meu problema de alergia a medicamentos	1	2	3	4	5
15	A ideia de tomar um medicamento deixa-me ansioso	1	2	3	4	5

## Abbreviations

DHR: Drug hypersensitivity reactions
HRQoL: Health-related quality of life
DrHy-Q: Drug Hypersensitivity Quality of Life Questionnaire
PROs: Patient-Reported Outcomes
ICC: Intra-class correlation coefficient
EFA: Exploratory factor analysis
PGWBI: Psychological General Well-being Index
ENDA: European Network of Drug Allergy
DPT: Drug provocation test
AICC: Akaike Information Criterion Corrected
NSAID: Nonsteroidal Anti-inflammatory drugs
ßL: Beta-lactam
RCM: Radiocontrast media
PPI: Proton pomp inhibitors
MPE: Maculopapular exanthema
SCARSs: Severe adverse cutaneous reactions
